# Nurse leaders' work‐related well‐being—Relationships to a superior's transformational leadership style and structural empowerment

**DOI:** 10.1111/jonm.13806

**Published:** 2022-10-10

**Authors:** Milja Niinihuhta, Anja Terkamo‐Moisio, Tarja Kvist, Arja Häggman‐Laitila

**Affiliations:** ^1^ Department of Nursing Science University of Eastern Finland Kuopio Finland; ^2^ City of Helsinki, Social and Health Care Helsinki Finland

**Keywords:** empowerment, nurse leader, nurse manager, transformational leadership style, work‐related well‐being

## Abstract

**Aims:**

To describe how nurse leaders' work‐related well‐being is related to a superior's transformational leadership style and structural empowerment.

**Background:**

The demanding role of nurse leader means that these professionals experience emotional exhaustion and challenges with work–life balance. They can also be influenced by the leadership style of their own superiors.

**Methods:**

A cross‐sectional questionnaire using two internationally validated scales, namely, the Transformational Leadership Scale and Conditions For Work Effectiveness Questionnaire‐II, was used. Statistical methods were applied during data analysis.

**Results:**

A total of 155 nurse leaders participated completed the questionnaire. The participants' work‐related well‐being scores ranged from 8 to 10. The participants felt that their superiors employ transformational leadership. The dimension of feedback and rewards received the lowest scores, whereas the nurse leaders reported moderate overall empowerment levels. A nurse leader's work‐related well‐being was positively correlated with structural empowerment and their superior's leadership style.

**Conclusions:**

Despite the fact that nurse leaders reported relatively high levels of work‐related well‐being, more attention should be paid on the feedback and rewards and on the support of superiors as they positively influence the work‐related well‐being.

**Implications for Nursing Management:**

Transformational leadership should be supported in organisations and through education as it strengthens work‐related well‐being and structural empowerment of nurse leaders.

## 1 BACKROUND

Work‐related well‐being can be conceptualised in numerous ways in the context of a workplace depending on discipline, organisation and country, among others. Furthermore, this type of well‐being is a highly complex concept that includes social, physical, psychological and emotional factors that can exist both inside and outside of the workplace. As an example, assessments of workplace well‐being solely focus on mental health aspects, whereas other measures incorporate work‐specific and personal characteristics (Buffet et al., 2013; Herttuala et al., 2020). In this study, work‐related well‐being is considered a subjective, comprehensive experience of one's physical, psychological, social and emotional state.

In a recent meta‐analysis, Membrive‐Jimenez et al. (2020) stated that the prevalence of emotional exhaustion—which negatively influences work‐related well‐being—can reach 29% among nurse leaders. This is unsurprising, as the demanding role of nurse leader can lead to stress and burnout, a risk which is exacerbated by unclear expectations of the leadership role (Coogan & Hampton, 2020) and challenges arising from staff shortages (Joslin & Joslin, 2021). Nurse leaders face a highly demanding task, as they are expected to look after the well‐being of their staff, provide direction for ward‐ and organisational‐level actions, be present, and behave as role models (Coogan & Hampton, 2020; Keisu et al., 2018; Udod et al., 2021). Moreover, a nurse leader's performance can significantly influence patient outcomes and employees' work‐related well‐being (Adriaenssens et al., 2017; Membrive‐Jimenez et al., 2020). Furthermore, nurse leaders across all organisational levels face challenges with work–life balance (Kelly et al., 2019). They are exposed to stressful situations on a daily basis, which requires high levels of tolerance and resilience (Hampton & Rayens, 2019). This stress can—understandably—affect a leader's individual work‐related well‐being. For this reason, nurse leaders need support from their organisation and superiors, which is recognised as a factor that enhances nurse leaders' work‐related well‐being (Coogan & Hampton, 2020; Herttuala et al., 2020; Trinchero et al., 2014). In this current study, the nurse leaders' levels and roles are differentiated so that leaders are considered persons who take the first‐line responsibility of the conduction of the nursing at the wards and superiors who are the principal chiefs of the leaders are working at the middle and upper level of the organisations taking responsibility of strategic planning of nursing.

It has been suggested that a nurse leader's control over their work and decision authority are factors that decrease the development of burnout and nurse leaders' turnover intentions. In contrast, poor leadership and a lack of autonomy may contribute to nurse leaders' burnout, whereas recognition, rewards and acknowledgement have been found to enhance work‐related well‐being (Kelly et al., 2019). In their study, Adriaenssens et al. (2017) found that nurse leaders who did not receive adequate support from their superiors showed significantly higher intention to leave than other participants. Labrague (2020) in turn found that the intentions to leave can be reversed especially by transformational leadership, positive organisational culture and trust in nurse directors. From these perspectives, the leadership style of nurse leaders' superiors is crucial to the working atmosphere of nurse leaders.

In particular, the transformational leadership style has been found to positively influence nurses' work‐related well‐being (Cummings et al., 2018; Kaffashpoor & Sadeghian, 2020). Nurse leaders' leadership styles, along with the effect on work‐related well‐being among nurses, have been extensively studied, with most research focussing on the transformational, ethical, transactional and laissez‐faire leadership styles (Niinihuhta & Häggman‐Laitila, 2022). However, there is limited evidence of how nurse leaders' superiors' leadership styles and nurse leaders' work‐related well‐being are related. Transformational leadership is a relationally focussed leadership style that can be characterised by the way a nurse leader supports the growth of employees and coaches them to achieve their goals. Transformational leaders exercise value‐driven, visionary and intellectually stimulating behaviours to establish an equal relationship with employees. As such, transformational nurse leaders challenge employees to participate in decision‐making and problem solving. (Ebrahimzade et al., 2015; Munir et al., 2012; Pishgooie et al., 2018; Sabbah et al., 2020).

Nurse leaders' empowerment has been identified as a further predictor of their job strain and turnover intentions (Wing et al., 2015). Empowerment, much like work‐related well‐being, is a multidimensional concept with various definitions (Abel & Hand, 2018). In the context of health care, empowerment has been described as the process of identifying and removing disempowering factors to improve nurse leaders' self‐efficacy (Fragkos et al., 2020). Empowerment can also be seen as the potential for gaining power or promoting nurse leaders' skills to advance positive changes in the work environment (Moura et al., 2020). Leadership has been shown to play an important role in the creation of empowering conditions at an organisation; thus, nurse leaders need to feel empowered to succeed in their demanding work (Trus et al., 2017).

One way to address empowerment is structural empowerment, that includes access to opportunity, information, support and resources in the workplace (Fragkos et al., 2020; Garcia‐Sierra & Fernandez‐Castro, 2018; Zhang et al., 2018). Access to opportunity describes the availability of options for professional development and enhancing one's expertise, whereas access to information involves both formal and informal knowledge and is crucial for improving effectiveness at work. Feedback, advice and guidance from supervisors and other colleagues fall under access to support, whereas access to resources includes the materials, time and means needed to perform work tasks (Garcia‐Sierra & Fernandez‐Castro, 2018; Orgambidez & Almeida, 2019). Structural empowerment also includes formal and informal power. The former involves rewards for innovation, visibility and flexibility, whereas the latter comprises collaboration with other professionals and seeking advice from peers (Laschinger et al., 2001). Structural empowerment has previously been associated with positive outcomes among nurses, for example, low burnout levels along with increased motivation, intention to stay and organisational commitment (Fragkos et al., 2020; Khan et al., 2018; Yürümezoğlu & Kocaman, 2019). In addition, Laschinger et al. (2012) associated senior nurse leaders' transformational leadership with the structural empowerment of both middle and first‐line nurse leaders.

There is extensive knowledge about how nurses' work‐related well‐being is associated with structural empowerment and their leader's leadership style. For example, it is well known that nurses who work in an environment with strong structural empowerment and relational leadership styles show high levels of work‐related well‐being (Cummings et al., 2018; Laschinger et al., 2013; Niinihuhta & Häggman‐Laitila, 2022) By contrast, the associations between nurse leaders' work‐related well‐being and perceived structural empowerment and their superiors' leadership style have not studied, to the best of our knowledge, in the recent decade. Only one article (Laschinger et al., 2012) adopted the nurse leader's perspective when reporting the associations between transformational leadership and structural empowerment, and this was published 10 years ago. A systematic review that included nine studies that solely focussed on nurse leaders' work‐related empowerment was published in the same year; the review reported a negative correlation between empowerment and emotional exhaustion and perceived health (Trus et al., 2012), and both factors that are associated with work‐related well‐being. The presented research focusses on the research gaps identified above of the relations concerning nurse leaders' work‐related well‐being, their experiences of structural empowerment and their superiors' leadership styles.

## 2 METHODS

### 2.1 Aim

The aim of the study was to describe how nurse leaders' work‐related well‐being is related to their superior's transformational leadership style and their experiences of structural empowerment.

The research questions were as follows:

1. How are nurse leaders assessing their overall work‐related well‐being?
2. Do nurse leaders feel that their superiors exercise transformational leadership?
3. To what extent do nurse leaders experience structural empowerment at their work?
4. How are a superior's transformational leadership style and structural empowerment related to nurse leaders' work‐related well‐being?

### 2.2 Study design

This research employed a cross‐sectional design.

### 2.3 Participants and recruitment

The participants of the study were nurse leaders working in one large organisation in Finland. The organisation provides outpatient and inpatient social and health care services to over 600,000 inhabitants and has over 15,000 employees. As such, it can be stated that the participants are working in a challenging environment. At the time of the research, the organisation was in the middle of a large renovation which aimed to integrate the social and health services (e.g. Tynkkynen et al., 2016), as well as challenged by a growing shortage of nurses.

The inclusion criteria were that the participant was a nurse leader and working in a unit providing 24‐hour care. Nurse leaders (*N* = 350) from acute care facilities, home hospital units, inpatient wards, mental health and substance abuse facilities, elderly services units, emergency departments and rehabilitation were invited to participate in the study. Prior to data collection, a power analysis was conducted using RAO software (McDonald, 2014). Based on the calculation, a sample size of 153 from a population size of 350 was needed to ensure a 5% margin of error and 90% confidence level. An information letter with a link to the electronic questionnaire was first sent to a contact person at the health care organisation, who then sent it via email to all of the nurse leaders who met the inclusion criteria.

### 2.4 Data collection and instruments

The data were collected anonymously from December 2015 to May 2016 using an electronic questionnaire that included demographic questions and two international instruments, namely, the Transformational Leadership Scale (TLS) and the Conditions For Work Effectiveness Questionnaire II (CWEQ‐II). Prior to data collection, a pilot study was conducted among 14 Finnish nurse leaders; the results of the pilot study did not indicate that any changes to the questionnaire were warranted. The demographic questions concerned the participant's age, gender, current title, current division and workplace. Furthermore, the participants were asked about their education, employment relationship and number of subordinates. The participants also assessed their well‐being at work using a scale that ranged from 4 (weakest) to 10 (strongest).

The TLS was designed to measure nurses' perceptions of nurse leader practices (Eneh et al., 2012; Kvist et al., 2019, 2013; Stevanin et al., 2020). The TLS includes a total of 49 questions across five different subscales: leadership ethics (14 questions); managing nursing process (16 questions); feedback and rewards (6 questions); professional development (7 questions); and characteristics of superiors (6 questions out of 11 questions from the original instrument). Respondents score each item using a 5‐point Likert scale. Previous studies have reported Cronbach's alpha values ranging from .88 to .97 for the scale (Eneh et al., 2012; Kvist et al., 2013).

The CWEQ‐II is based on Kanter's theory of structural empowerment (1977), which considers workplace structures that support employees to succeed as empowering. The CWEQ‐II operationalises the six sub‐concepts of Kanter's theory (Opportunity, Information, Support, Resources, Job Activities Scale [JAS] and Organisational Relationships Scale [ORS]), as well as includes two questions about global empowerment, which serve as a validation index (Laschinger, 2012). The questionnaire includes 21 items that are scored on a 5‐point Likert scale. Previous studies have reported Cronbach's alpha values ranging from .78 to .94 for the scale (Taan et al., 2020; Trus et al., 2017).

During data collection, we also gathered work‐related well‐being data from the target group using four internationally validated scales that focussed on work engagement, working conditions, sense of coherence and burnout. These results have been reported in Niinihuhta et al. (2022). When responding to these scales, the nurse leaders assessed their working conditions along with the associations between work, health and productivity, leadership skills, mastery of work and role expectations, ability and capability to face problems, and stressors.

### 2.5 Data analysis

The data were analysed using SPSS 27 (IBM Corporation, Armonk, NY) for Windows. Descriptive statistics were employed to present the participants' demographics. In further analyses, the participant age was recoded into four groups of approximately the same size (30–46, 47–51, 52–56, and 57–66 years). In addition, the number of employees a participant had was recoded into three groups (>20, 20–39, and 40–99), whereas leadership level was recoded into two groups (manager and director levels). Lastly, work experience in health care was recoded into five groups (5–19, 20–24, 25–29, 30–35, and 36–42 years), whereas experience in leadership was recoded into four groups (0–5, 6–10, 11–15, and 16–30 years).

Sum variables were calculated based on previously published descriptions of the instrument structures (Eneh et al., 2012; Laschinger, 2012). Relationships between variables were examined with Spearman's correlations, with *r* values ≥ .3 indicating intermediate correlation and *r* values between .31 and .5 indicating strong correlation (Field, 2013). The non‐normality of the data was confirmed with the Kolmogorov–Smirnov test (Field, 2013). The threshold for statistical significance was set as *p* < .05. The internal consistency of the calculated sum variables was examined by calculating Cronbach's alpha values, with values >.7 regarded as good, whereas values between .6– and .7 were regarded as acceptable (Field, 2013).

### 2.6 Ethical considerations

Based on Finnish legislation, this study—which followed the ethical guidelines for research—did not require ethical approval. However, research approval was obtained from the studied organisation because of their requirements. The participants were informed about the study in written form and were also given the possibility to ask additional questions from the researcher prior to confirming their voluntary participation (The World Medical Association, 2018). All of the data were collected anonymously and stored in a secure file that was only accessible to members of the research group (Nordic Nurses Federation, 2003). Permission to use both scales was asked for and received.

## 3 RESULTS

### 3.1 Participants

A total of 155 nurse leaders participated in this study (Table 1), which reflects a response rate of 44%. Most of the participants were female (97%), and the mean age was 51 years (range 32–66 years). Most of the participants (39%) had a degree from a university of applied sciences, followed by college (33%) and university degrees (28%). The participants had, on average, 24 years (range 5–41 years) of experience in nursing, and 10 years (range 1–30 years) of experience in nursing leadership. Most of the participants worked as nurse managers (89%), with almost all having a permanent contract (94%). Over half of the participants (58%) were in charge of 20–39 subordinates. Most of the participants worked in acute care, rehabilitation or elderly services units (88%), followed by mental health and substance abuse facilities (9%) and family and social services facilities (3%).

**TABLE 1:** Participants demographics (*n* = 155)

	*f*	%
Gender (*n* = 152)		
Male	4	2.6
Female	148	97.4
Age in years (*n* = 151)		
30–46	36	23.8
47–51	47	31.1
52–56	32	21.2
57–66	36	23.8
Level of education (*n* = 154)		
College	51	33.1
University of applied sciences	60	39.0
University degree	43	27.9
Managerial level (*n* = 153)		
Manager	136	88.9
Director	17	11.1
Workplace (*n* = 155)		
Acute care, rehabilitation, or elderly services units	136	87.7
Mental health and substance abuse facilities	14	9.0
Family and social services facilities	4	2.6
Number of subordinates (*n* = 137)		
> 20	45	32.8
20–39	79	57.7
40–99	13	9.5
Experience in healthcare in years (*n* = 151)		
5–19	33	21.9
20–24	36	23.8
25–29	32	21.2
30–35	34	22.5
36–41	16	10.6
Managerial experience in years (*n* = 145)		
0–5	40	27.6
6–10	61	42.1
11–15	18	12.4
16–30	26	17.9

### 3.2 Nurse leaders**'** work‐related well‐being

The participants reported work‐related well‐being scores between 8 and 10 (mean 8.12, SD 1.1). A minority (15.8%) of the nurse leaders rated their work‐related well‐being at the highest possible level (a score of 10), with most of the participants (43.9%) reporting a score of 8 for work‐related well‐being.

### 3.3 Transformational leadership among nurse leaders' superiors

When asked about their superior's transformational leadership style, the participants reported scores between 1.1 and 5.0 (mean 4.0, SD 0.84). The dimension of TLS which received the highest mean score (4.11, SD 0.80; Table 2) was professional development, followed by leadership ethics (mean 4.07, SD 0.90), managing nursing process (mean 4.00, SD 0.86) and superiors (mean 3.96, SD 0.97). The participants rated feedback and rewards (mean 3.64, SD 1.00) as the weakest dimension of TLS.

**TABLE 2:** Transformational leadership scale results (*n* = 155)

Dimension	No. of items	*n*	Min	Max	Mean	SD	*α*
Leadership ethics	14	146	1.00	5.00	4.07	0.90	.96
Managing nursing process	16	137	1.06	5.00	4.00	0.86	.97
Feedback and rewards	6	144	1.17	5.00	3.64	1.00	.92
Professional development	7	141	1.14	5.00	4.11	0.82	.90
Top manager	6	147	1.00	5.00	3.96	0.97	.93

*α* = Cronbach's alpha.

### 3.4 Nurse leaders' structural empowerment

The participating nurse leaders reported a moderate level (mean 21.5, SD 3.05) of overall empowerment. The strongest individual dimension of structural empowerment (Table 3) was opportunity (mean 4.20, SD 0.69), followed by information (mean 4.05, SD 0.71) and ORS (mean 3.74, SD 0.63). The three weakest dimensions of this scale were resources (mean 3.33, SD 0.82), JAS (mean 3.18, SD 0.77), and support (mean 3.05, SD0.98).

**TABLE 3:** Participants structural empowerment (*n* = 155)

Dimension	No. of items	*n*	Min	Max	Mean	SD	*α*
Opportunity	3	148	2.33	5.00	4.20	0.69	.84
Information	3	145	2.00	5.00	4.05	0.71	.78
Support	3	145	1.00	5.00	3.05	0.98	.87
Resources	3	147	1.00	5.00	3.33	0.82	.78
JAS	3	147	1.00	5.00	3.18	0.77	.73
ORS	4	145	1.25	5.00	3.74	0.63	.68

*α* = Cronbach's alpha.

### 3.5 Relationships between nurse leaders' work‐related well‐being and other factors

Nurse leaders' work‐related well‐being was found to be positively correlated with the extent to which their superiors employ transformational leadership (Table 4). This indicates that the stronger the transformational leadership style the own superior has, the stronger is the work‐related well‐being of the nurse leader. Further positive correlations were found between a nurse leader's work‐related well‐being and perceived structural empowerment (Table 4); in other words, nurse leaders who experience sufficient levels of structural empowerment will experience high work‐related well‐being.

**TABLE 4:** Correlations between work‐related well‐being and associated factors

	Work‐related well‐being	
	*r*	*p*
Transformational leadership style	**.319**	**<.001**
Leadership ethics	.403	<.001
Managing nursing process	.319	<.001
Feedback and rewards	.319	<.001
Professional development	.312	<.001
Top manager	.289	<.001
Structural empowerment	**.391**	**<.001**
Information	.248	.003
Support	.295	<.001
Resources	.342	<.001
JAS	.222	.007
ORS	.237	.004

*Notes*: JAS, job activities scale; ORS, organisational relationships scale.

A closer examination of the relationship between structural empowerment and TLS revealed strong statistically significant correlations (Table 5). The access to support correlated most strongly with the dimension of TLS. Although the informal power showed the weakest correlations.

**TABLE 5:** Correlations between structural empowerment and transformational leadership style

	TLS leadership ethics	TLS managing nursing process	TLS feedback and rewards	TLS professional development	TLS nursing director
SE opportunity	*r* = .228 *p* < .001	*r* = .349 *p* < .001	*r* = .382 *p* < .001	*r* = .309 *p* < .001	*r* = .261 *p* < .001
SE information	*r* = .353 *p* < .001	*r* = .363 *p* < .001	*r* = .440 *p* < .001	*r* = .431 *p* < .001	*r* = .279 *p* < .001
SE support	*r* = .560 *p* < .001	*r* = .613 *p* < .001	*r* = .695 *p* < .001	*r* = .594 *p* < .001	*r* = .447 *p* < .001
SE resources	*r* = .472 *p* < .001	*r* = .419 *p* < .001	*r* = .340 *p* < .001	*r* = .454 *p* < .001	*r* = .381 *p* < .001
SE JAS	*r* = .409 *p* < .001	*r* = .449 *p* < .001	*r* = .485 *p* < .001	*r* = .438 *p* < .001	*r* = .385 *p* < .001
SE ORS	*r* = .221 *p* < .001	*r* = .227 *p* < .001	*r* = .255 *p* < .001	*r* = .263 *p* < .001	*r* = .135 *p* = .109
SE GE	*r* = .546 *p* < .001	*r* = .506 *p* < .001	*r* = .574 *p* < .001	*r* = .506 *p* < .001	*r* = .409 *p* < .001
SE total	*r* = .581 *p* < .001	*r* = .643 *p* < .001	*r* = .726 *p* < .001	*r* = .665 *p* < .001	*r* = .478 *p* < .001

*Notes*: SE, Structural empowerment; TLS, transformational leadership style; JAS, job activities scale; ORS, organisational relationships scale.

## 4 DISCUSSION

The participants in this study assessed their work‐related well‐being to be at a relatively high level. This is a positive finding, especially because stress and burnout among nurse leaders has been acknowledged as a growing problem (Djukic et al., 2017; Remegio et al., 2021; Saifman & Sherman, 2019). Work‐related well‐being can be considered as a result of several organisational and individual factors (Buffet et al., 2013); therefore, it should be assessed on a multifactorial level by including aspects such as structural empowerment and leadership styles (Niinihuhta & Häggman‐Laitila, 2022). In addition, previous studies have revealed that work‐related well‐being is associated with stress (Liu et al., 2019; Niinihuhta et al., 2022), working conditions, experienced status of health, sense of coherence (Niinihuhta et al., (2022) and leadership skills (Niinihuhta & Häggman‐Laitila, 2022) underlining the need to take also these into the account in the future studies.

Most of the participants felt that their superiors employed a transformational leadership style. In a prior study, Kvist et al. (2013) found nursing leadership in Finnish hospitals to be administrative rather than transformational; this led the researchers to conclude that there is a need for further efforts to achieve transformational leadership across all organisational levels. Compared our results with the Kvist et al. (2013), the nurse leaders seem more likely to assess their superiors' leadership style as transformational than nurses. One example to explain this could be nurse leaders' deeper understanding on their superiors' job descriptions than nurses have of their leaders. Another possibility could be that there is more interaction between nurse leaders and their superiors than between nurses and their leaders. Also, the higher level of education among nurse leaders compared to nurses may partly explain the difference; however, this should be studied further.

In the current study, the dimension of feedback and rewards was the weakest component of a superior's leaderships style; this result agreed with what was reported by Kvist et al. (2013). Seitovirta et al. (2018) also found in their study focussing on rewarding that nurses have been generally unsatisfied with it. Although generational differences in nurses' satisfaction with feedback and rewards have been noted, this aspect of leadership exerts a positive influence on work‐related well‐being among all nurses through outcomes, stability and performance (Seitovirta et al., 2018; Stevanin et al., 2020). In addition, support, rewards and acknowledgement enhance nurse leaders' work‐related well‐being and reduce their turnover intentions (Kelly et al., 2019; Miller, 2020). Thus, this aspect of leadership warrants future attention, for example, in leadership education.

Health care organizations should consider interventions that would develop superiors' leadership styles, because these have a clear connection on nurses' and nurse leaders' work‐related well‐being. More specifically, research could provide insight into how leadership style affects the whole team and the workplace culture, rather than just individual relationships between nurses, nurse leaders and their superiors (e.g. Cummings et al., 2018).

The participants' experiences of moderate levels of structural empowerment are in line with the findings of previous research (Connolly et al., 2018; Moura et al., 2020; Trus et al., 2018, 2017). Researchers have also previously acknowledged relationships between structural empowerment and factors associated with work‐related well‐being, for example, lower work stress, emotional burnout and organisational commitment (Fragkos et al., 2020; Jafari et al., 2021; Zhang et al., 2018). In a recent meta‐analysis Zhang et al. (2018), stated that nurses' structural empowerment is negatively correlated with emotional exhaustion, and that this challenge can be alleviated by enhanced structural empowerment. Structural empowerment has also been linked to organisation's success (e.g. de Almeida et al., 2017; Goedhart et al., 2017) and positive organisational consequences such as adaptive resilience, work engagement and retention (Abel & Hand, 2018), and thus, future research should focus on how each dimension of structural empowerment can be further enhanced.

MacPhee et al. (2012) stated that nurse leaders who use structural empowerment strategies create a high‐quality, safe work environments that empower other employees. Leadership training could offer the means for strengthening structural empowerment at a health care organisation (MacPhee et al., 2012), and research into these types of interventions is relevant because structural empowerment is closely connected to work performance and decreased turnover of employees (Fragkos et al., 2020). Despite extensive research on structural empowerment, Häggman‐Laitila and Romppanen (2018) did not find any interventions related to work‐related well‐being that were based on structural empowerment. Therefore, this topic requires additional research.

Our results revealed that access to support was the weakest dimension of structural empowerment. It is difficult to compare this result with previous reports, because only a limited number of studies have investigated nurse leaders' structural empowerment. However, previous studies with a focus on nurses have revealed low levels of access to support (Connolly et al., 2018; Moura et al., 2020). Support can be received from one's superior, and also from peers and subordinates. The support nurse leaders receive from their organisation and superiors has been recognised to enhance nurse leaders' work‐related well‐being (Coogan & Hampton, 2020; Herttuala et al., 2020; Trinchero et al., 2014), whereas access to support can enable autonomous decision‐making (Moura et al., 2020). Future research should identify the type of support that nurse leaders find supportive. This evidence could help clarify the approaches through which organisations can enhance nurse leaders' autonomy, involvement, activity and trust. Deeper knowledge about the development of support processes in organisations is also needed. For example, Penconek et al. (2021) suggested that evaluating the work loads of nurse leaders, offering clinical and administrative support, and developing co‐managing and transformational leadership could all lead to higher job satisfaction.

### 4.1 Strengths and limitations

All of the participants were from the same region of Finland, which may be considered a limitation. Furthermore, only a minority of the participants were nurse leaders working at upper leadership level in the organisation; thus, little can be stated about the work‐related well‐being of them. This indicates that there is a need for future research on this topic. A strength of the research was that the study was conducted in a large organisation that included various working environments. Moreover, the response rate in this study was high (44%), which strengthens the validity of the results. The choice to apply internationally validated instruments strengthens the trustworthiness of the research. In addition, the Cronbach's alpha values calculated in this study varied from .68 to .97, which demonstrates acceptable internal consistency. The associations between work‐related well‐being and structural empowerment were based on nurse leaders' self‐assessments, which reduces the validity of the study. However, the findings about how transformational leadership is linked to work‐related well‐being were based on nurse leaders' ratings of their superiors' leadership styles, which enhances the validity of the study.

## 5 CONCLUSIONS

This study focussed on the research gap concerning how perceived structural empowerment and a superior's transformational leadership style affect nurse leaders' work‐related well‐being. The current study had three main findings. First, the participating nurse leaders had relatively high work‐related well‐being. This shows that they were provided adequate resources despite working in challenging and stressful environment. Second, the participants felt that their superiors exercised transformational leadership. The weakest dimension of this leadership style was feedback and rewards, and organisations should specifically focus on this dimension when developing transformational leadership among leaders. Third, there was a discrepancy between work‐related well‐being and structural empowerment, with the nurse leaders perceiving moderate levels of the latter. It is crucial for organisations to develop all dimensions of structural empowerment, with the dimension of support particularly important.

Health care organisations should pay more attention to support, feedback and rewards across all organisational levels if they aspire to improve work‐related well‐being, which is critical for tackling staff shortages and retention of nurse leaders. Organisations should also promote and increase the use of transformational leadership to strengthen nurse leaders' structural empowerment, and thus, work‐related well‐being.

### 5.1 Implications for nursing management

The work of a nurse leader can be extremely challenging and demanding, potentially causing significant stress among those with limited experience in leadership. This will naturally have an effect on their work‐related well‐being. Organisations can tackle this, for example, by providing flexible working hours and by helping nurse leaders maintain a healthy work–life balance. Continuous leadership training ensures that the challenges which nurse leaders experience are in line with their competences, that is, providing constructive feedback. Organizations should ensure that leaders in every organizational level employ transformational leadership, as well as enhance structural empowerment among nurse leaders, in a bid to strengthen leaders' and nurses' work‐related well‐being (Hampton & Rayens, 2019).

The existing literature demonstrates that nurses and nurse leaders only perceive moderate levels of structural empowerment (e.g. Eskandari et al., 2017; Jafari et al., 2021; Regan et al., 2016; Trus et al., 2018) Hence, organizations should continue to strive towards improved structural empowerment, which can reduce costs and improve the quality of services (Jafari et al., 2021). Moreover, the strong correlation between structural empowerment and work‐related well‐being shown in this study indicates a strong need for policies and structures that enhance nurses' and nurse leaders' structural empowerment in health care organizations.

Organizations can use the results of presented research to focus the assessment and development of the weaker areas of the leadership, for example feedback and support, to enhance their nurse leaders' work‐related well‐being. Organisations should invest in nurse leaders who can create empowering environments from the bottom to the top of the organisation (Hampton & Rayens, 2019; Laschinger et al., 2012). The organisational culture and practices should be supportive of both transformational and empowering leadership (Khan et al., 2018). Nurse leaders who are supported and empowered by their superiors will, in turn, direct the same support and empowerment to their staff nurses (Laschinger et al., 2012). Health care organisations can positively affect nurse leaders' retention, turnover intentions, empowerment and work‐related well‐being by offering mentoring, coaching and leadership training (Labrague, 2020; Laschinger et al., 2012).

## CONFLICTS OF INTEREST

The authors declare that they have no competing interests.

## ETHICAL APPROVAL STATEMENT

Based on the Finnish legislation, this study—which followed the ethical guidelines for research—did not require ethical approval.

## Data Availability

The data that support the findings of this study are available from the corresponding author upon reasonable request.
